# Bone of contention: The applicability of the Greulich–Pyle method for skeletal age assessment in South Africa

**DOI:** 10.4102/sajr.v22i1.1348

**Published:** 2018-08-08

**Authors:** Dashnee Govender, Matthew Goodier

**Affiliations:** 1 Department of Radiology, Grey’s Hospital, University of KwaZulu-Natal, South Africa

## Abstract

**Background:**

The Greulich–Pyle (GP) method is one of the most commonly used radiographic techniques for skeletal age (SA) determination. The applicability of this method for populations outside of the United States has been questioned and this has been investigated in several recent studies around the world. Only limited data are available on the accuracy of the GP method for a South African population.

**Objective:**

To determine the accuracy and reliability of the GP method in a population from KwaZulu-Natal, South Africa.

**Method:**

A retrospective study was performed using a digital database consisting of 102 hand-wrist radiographs. The SA was estimated by two readers with different levels of experience, using the GP method. Differences between estimated SA and chronological age (CA) were analysed.

**Results:**

Skeletal age determined with the GP method was found to significantly differ from CA. For the population as a whole, the GP method underestimated age. The greatest mean underestimation between SA and CA was 11.5 ± 17 months and 7.4 ± 13.2 months for the 10.1 to 15 year age groups in male and female patients, respectively. The method was found to have excellent inter- and intra-observer reliability.

**Conclusion:**

The GP method generally significantly underestimates age for both genders. Overestimation is also possible with individuals as young as 16 years old found by the method to be skeletally mature. Until new SA assessment tools are developed for South Africa, use of supplementary means of determination of SA should be considered, especially in medico-legal cases.

## Introduction

Skeletal maturity assessment is used in a variety of settings, including medical, forensic, medico-legal and for sporting reasons.^1,2,3^ There are various radiographic methods of skeletal age (SA) assessment; however, a commonly used technique is to compare a single hand-wrist radiograph with a standard reference.^4^ The Greulich–Pyle (GP) method, because of its simplicity, is one of the most frequently utilised methods worldwide.^5,6^ The major alternative technique utilising a hand-wrist radiograph is the Tanner–Whitehouse 2 (TW2) method.^7^ Assessment of the degree of ossification of the medial clavicular epiphysis^8^ or evaluation of the ossification and fusion of the iliac crest apophyses (Risser staging system) are useful in certain specific situations. Methods that utilise magnetic resonance imaging or ultrasound have also been described but are not widely used.^9,10^ Odontological evaluation is a non-radiological technique for estimating SA.^11^

Greulich and Pyle published their ‘Radiographic atlas of skeletal development of the hand and the wrist’ in 1959. This atlas contains reference images of male and female standards of the left wrist and hand from birth until 19 years for male and 18 years for female patients. The standards are based on a population of ‘Caucasian’ children, ‘above average in economic and educational status’, residing in the United States, using measurements compiled in the 1930s.^12^ SA is determined by a comparison between the left hand-wrist radiograph of the subject to the nearest matching reference radiograph.

The radiographic standards used in the GP method were based on a narrowly defined population, which was both genetically and socio-economically homogeneous. It is far from clear if these standards of skeletal maturation using data from the first half of the twentieth century can be applied to current populations that differ geographically, genetically and socio-economically from the GP reference population. Indeed, studies performed in numerous countries have sought to assess the accuracy of the GP atlas in various populations.^13,14,15,16,17,18,19,20,21^ Some studies have concluded that the GP atlas is applicable to the studied populations.^19,20^ However, most have questioned the accuracy of the GP method, particularly in less developed countries such as Turkey, Malawi and South Africa.^13,14,15,16,18^

A further factor that may affect the utility of a given method in a less developed setting is the performance of the test in the hands of inexperienced readers. Groell et al. found a trend that suggests that the reliability of the bone age estimations was higher in experienced readers compared to radiology residents; however, the differences did not reach statistical significance.^19^ Chiang et al. failed to show a statistically significant difference between readers of different levels of experience.^21^

Despite the widespread use of this method, there is a scarcity of local research on the accuracy and reliability of the GP method. To date only one published study of the method, limited to a male population, has been performed in South Africa.^18^ Our study is the first to assess the accuracy and reliability of the GP method of skeletal assessment for both sexes in a South African population.

## Research methods and design

A quantitative, observational analytical, comparative cross-sectional study was conducted during May–June 2017, on 102 patients of both sexes, aged between 0 and 21 years, who had hand-wrist radiographs at Grey’s Hospital, Pietermaritzburg, South Africa, between January 2012 and August 2016.

**FIGURE 1:**
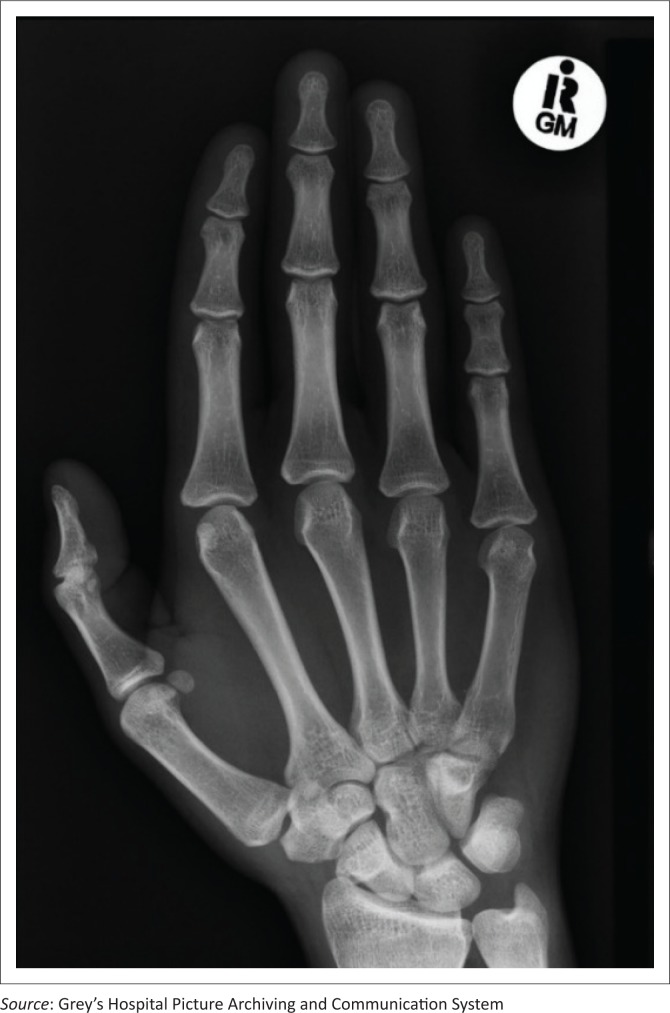
Digital hand-wrist radiograph (right) of a female subject with a chronological age of 20 years 8 months was matched to the GP standard image of a 17-year-old by both readers.

**FIGURE 2:**
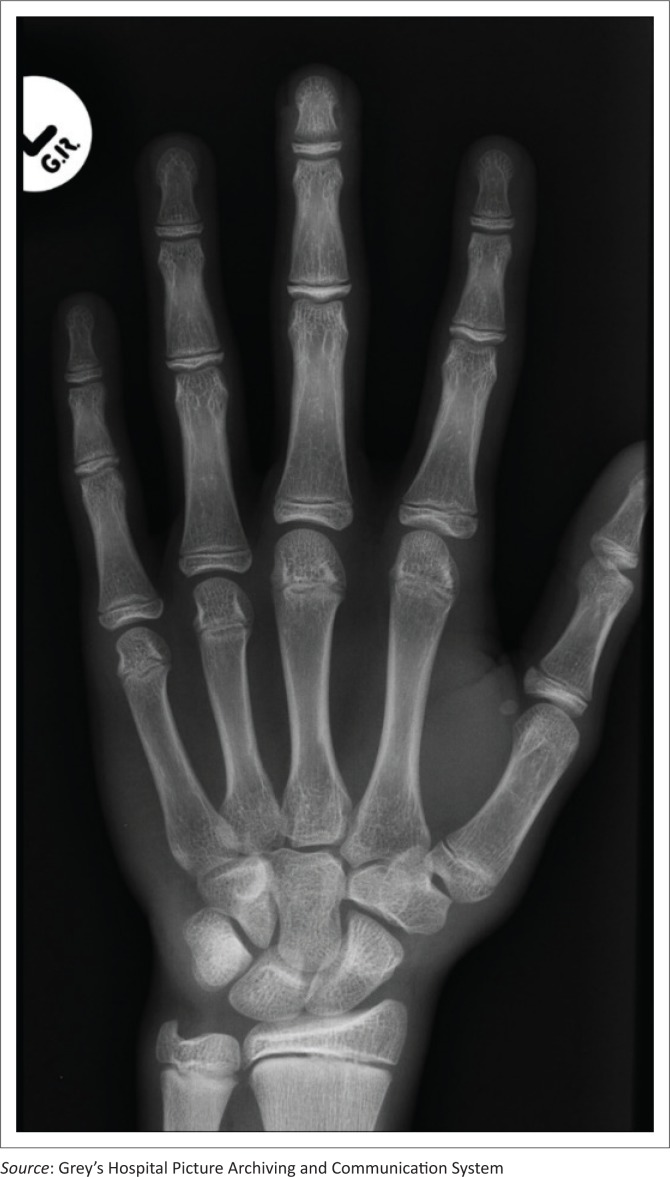
Digital hand-wrist radiograph (left) of a male subject with a chronological age of 15 years 10 months was matched to the GP standard image of a 14-year-old subject by both readers.

Digital hand-wrist images obtained by computed radiography were accessed from the Picture Archiving and Communication System (see Figures 1 and 2). Antero-posterior (AP) or postero-anterior (PA) views including the phalanges, metacarpals, carpals, distal ulna and radius were used. The GP atlas uses the left hand-wrist radiograph by convention as a standard for SA assessment. When images of both hands for the same patient were available, the left-sided radiograph was used. If only the right-sided radiograph was available, then this was used. All the radiographs included in the study were of outpatients who had been imaged in the emergency department for suspected trauma as identified by the history provided on the imaging request form. Radiographs performed as part of the work up of significant medical or systemic disorders were specifically excluded.

Poor quality radiographs (poor clarity, poor positioning and incomplete views) were excluded. Patients with fractures, congenital abnormalities, arthritis or any other obvious disease of the hands and wrist that could affect skeletal maturation or hinder determination of age were excluded.

Two readers of different levels of experience, a specialist radiologist with five years of experience and a radiology registrar in the first year of training, independently assessed the SA according to the GP method. The readers were blinded to the chronological age (CA) of each subject. In order to assess intra-observer reliability, following an interval of approximately five weeks after the initial assessment, a subset of 10 randomly selected radiographs from the sample were again reviewed independently by the same two readers, again blinded to the CA.

## Ethical considerations

This was a retrospective study of radiological records and images. Patient confidentiality was ensured by anonymisation of all patient data. Ethical approval was obtained from University of KwaZulu-Natal Biomedical Research Ethics Committee (BE006/17). Consent was obtained from Grey’s Hospital management.

## Results

**FIGURE 3:**
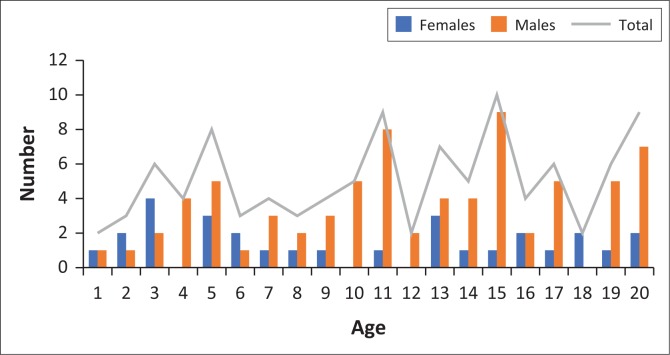
Age and sex distribution of the study population.

Figure 3 shows the age and sex distribution of the study population. Of note is the larger number of individuals from the older age groups and the male predominance of the study population.

**FIGURE 4:**
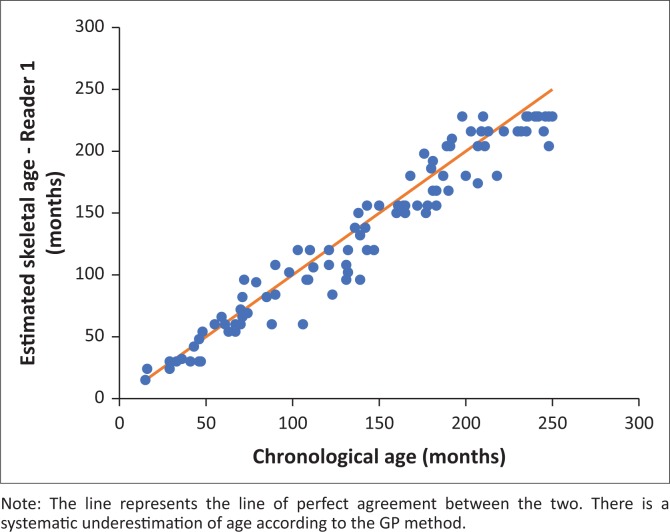
Scatterplot of the skeletal age estimated by Reader 1 (y-axis) and the chronological age (x-axis) in months (Pearson’s correlation coefficient = 0.973).

**FIGURE 5:**
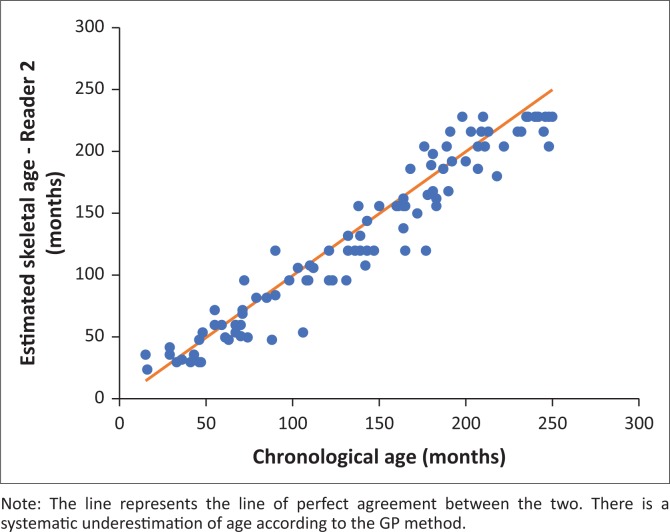
Scatterplot of the skeletal age estimated by Reader 2 (y-axis) and the chronological age (x-axis) in months (Pearson’s correlation coefficient = 0.966).

Figures 4 and 5 illustrate the linear correlation between SA as estimated by the GP method and CA for the reader. In the figures, Reader 1 refers to the radiology consultant and Reader 2 refers to the radiology registrar.

The intraclass correlation coefficient (ICC) was used to measure the consistency of measurements between the observers. An *F* test was used to test if the ICC was significantly different from 0. The ICC between the two reader’s estimates was 0.99 (*p* < 0.001). Therefore, correlation was excellent between the two readers (an ICC of > 0.75 is considered excellent).

Table 1 shows the difference between SA and CA estimations by Reader 1 for the population as a whole. A one-sample *t*-test was performed showing the difference was statistically significant (*p* < 0.001). Mean difference = mean CA - mean SA. The positive mean difference (7.4 ± 15.7) indicated an underestimate. Therefore, application of the GP method resulted in a statistically significant underestimation of age (*p* < 0.001).

**TABLE 1:** Whole population difference between the skeletal age and chronological age for Reader 1.

Age variables	*n*	Mean	SD	Min	Max
Estimated SA (months) – Reader 1	102	135.7	65.8	15	228
Chronological age (months)	102	143.1	67.9	15	250
Difference (CA − SA)	102	7.4	15.7	−30	46

CA, chronological age; SA, skeletal age; SD, standard deviation.

Following a similar methodological approach to that of Dembetembe et al.^18^ Tables 2 and 3 were divided into two sections of analysis. The first section deals with patients prior to age of expected skeletal maturity and the second section for patients at or after the age of expected skeletal maturity (18 and older in female patients and 19 years and older in male patients). This is because the estimated SA is no longer able to be correlated with CA after full skeletal maturity is reached and thus an increasing discrepancy between estimated SA and CA is expected to occur with increasing CA. Individuals after the expected age of skeletal maturity were thus presented separately.

**TABLE 2:** Group analysis of difference between the skeletal age and chronological age for Reader 1 for various ages in male patients only (*N* = 61).

Variable	Years	*n*	SA estimation		Difference (CA − SA)			
			Mean (years)	SD (years)	Mean (years)	Mean (months)	SD (years)	SD (months)
CA group	0.0–5.0	8	3.8	1.4	−0.3	−3.0	0.4	5.0
	5.1–10.0	14	6.7	1.9	0.5	6.0	1.5	17.8
	10.1–15.0	24	11.4	2.5	1.0	11.5	1.4	17.0
	15.1–19.0	15	16.0	1.8	0.3	3.2	1.5	18.1
Mean	-	-	9.5	1.9	0.4	4.4	1.2	14.5
For CA greater than Greulich–Pyle maturation age	19.1–21.0	12	18.8	0.5	1.3	15.1	0.4	4.8

CA, chronological age; SA, skeletal age; SD, standard deviation.

**TABLE 3:** Group analysis of difference between the skeletal age and chronological age for Reader 1 for various ages in female patients only (*N* = 24).

Variable	Years	*n*	Skeletal age estimation		Difference (CA − SA)			
			Mean (years)	SD (years)	Mean (years)	Mean (months)	SD (years)	SD (months)
CA group	0.0–5.0	7	2.5	0.7	0.6	6.7	0.6	7.8
	5.1–10.0	8	7.0	1.8	−0.2	−2.6	0.8	10.0
	10.1–15.0	5	12.9	0.2	0.6	7.4	1.1	13.2
	15.1–18.0	4	16.6	2.4	−0.1	−1.8	1.7	20.2
Mean	-	-	9.7	1.3	0.2	2.4	1.1	12.8
For CA greater than Greulich–Pyle maturation age	18.1–21.0	5	17.2	1.3	2.2	26.6	1.3	15.6

CA, chronological age; SA, skeletal age; SD, standard deviation.

Amongst male patients, the mean differences (CA − SA) ranged from an overestimation of 3 ± 5 months to an underestimation of 11.5 ± 17 months for the age categories ≤ 19 years. Overall, a mean underestimation of 4.4 ± 14.5 months was recorded for age categories ≤ 19 years.

Amongst female patients, the mean differences (CA minus SA) ranged from an overestimation of 1.8 ± 20.2 months to an underestimation of 7.4 ± 13.2 months for the age groups ≤ 18 years. Overall, a mean underestimation of 2.4 ± 12.8 months was noted for the age categories ≤ 18 years.

According to the GP atlas, skeletal maturity is reached at 18 years for women and at 19 years for men. Table 4 shows the number of female patients who had achieved full skeletal maturity in different age groups. There were two female patients in the study population with a CA of 18. Only one of these female patients was found to be both chronologically and skeletally 18 years old. The other chronologically 18-year-old was estimated to have a SA of 15. Interestingly, there were two female patients with a CA of 20, who were found to have SAs of 18 and 17. One female patient with a CA of 16 had a SA of 18 (i.e. had attained skeletal maturity). Similarly, one female patient with a CA of 17 had a SA of 18.

**TABLE 4:** The number and percentage of skeletally mature female patients per chronological age group.

CA groups	*N*	Individuals with complete epiphyseal fusion
≥ 16 to < 17	2	1
≥ 17 to < 18	1	1
≥ 18 to < 19	2	1
≥ 19 to < 20	1	1
≥ 20 to < 21	2	1

Note: Only the age groups in which skeletally mature female patients were found are shown.

CA, chronological age; *N*, total number of individuals in the age group.

Table 5 shows the number of male patients who had achieved full skeletal maturity in different age groups. Male patients who were both chronologically and skeletally 19 years old represent only 40% of the 19-year age group. Therefore, 60% of the 19-year-old male patients had not reached skeletal maturation. The remaining three male patients who had CAs of 19 years all had SAs of 18. There was one male patient with a CA of 16 who had a SA of 19 (i.e. attained skeletal maturity). One male patient with a CA of 17 had a SA of 19.

**TABLE 5:** The number and percentage of skeletally mature male patients per chronological age group.

CA groups	*N*	Individuals with complete epiphyseal fusion
≥ 16 to < 17	2	1
≥ 17 to < 18	5	1
≥ 18 to < 19	0	0
≥ 19 to < 20	5	2
≥ 20 to < 21	7	7

Note: Only the age groups in which skeletally mature male patients were found are shown.

CA, chronological age; *N*, Total number of individuals in the age group.

Pearson’s correlation coefficient (*r*) was calculated and a paired ***t***-test was performed to assess intra-observer reliability showing a strong correlation (Pearson *r* = 0.99) between the two readings performed five weeks apart with no statistical significant difference noted in the readings (*p* > 0.05).

## Discussion

In light of the uncertainty related to the applicability of the GP method in South Africa, we set out to investigate the accuracy and reliability of the method in a local population.

There was a distinct male predominance in the sample population. This was also a feature of similar previous studies.^16,17^ This is likely accounted for by an anticipated higher number of male patients in a population selected from referrals for suspected trauma. A similar reason explains the relative lack of very young patients < 5 years of age.

The reliability of the GP method in our study was acceptable with excellent inter- and intra-observer agreement. Similar findings occurred in previous studies.^19,21^ This is thought to be because of the simplicity of the GP method^6^ and is one of the reasons why the GP method is such a popular method of SA estimation method worldwide.^5^ Our study also found no significant difference between the two readers of differing levels of experience with good agreement between the consultant radiologist and the registrar. This suggests that the concept of SA estimation by means of comparison with radiographic standards is not only reliable but also intuitive and can be readily utilised even with little prior experience.

The most important findings of the study were the poor accuracy of the GP method. Overall, there was a statistically significant underestimation of age for both genders. The results were more variable in specific age groups, with age being on average overestimated in some age groups and underestimated in others.

Interestingly, the results of other studies performed in Africa have also suggested that the atlas underestimates age.^16,18^ The study performed in Malawi by Lewis et al. demonstrated a significant underestimation of SA in Malawian children when compared to the GP atlas reference radiographic standards.^16^ The study by Dembetembe et al. performed on an exclusive male population from the Western Cape province of South Africa also showed a consistent underestimation of age. In that study, epiphyseal fusion of the hand and the wrist was not often complete by 19 years, leading the authors to suggest that the onset of epiphyseal fusion occurs approximately two years later in male Africans. Internationally, studies performed in Brazil and Turkey showed that the GP method consistently overestimated age for both sexes.^13,22,23^

Of particular medico-legal and forensic interest, is the possibility of drawing erroneous conclusions of adulthood based on the finding of full skeletal maturity by radiographic methods. Such errors could result in children being considered by the legal system as adults and vice versa. This study found several instances of children under 18 years of age having attained full skeletal maturity as well as individuals 18 years and older with immature skeletons. These findings suggest that reliance on the GP method as evidence for age determination in defendants of uncertain age lacks a basis in the scientific literature.

The normal progress of skeletal maturation in children is a complex and multifactorial process. Our study population is different from the reference population originally studied by Greulich and Pyle in numerous ways. We studied a genetically heterogeneous population likely to be from diverse socio-economic circumstances. Acquired systemic disorders (e.g. human immunodeficiency virus [HIV] and tuberculosis [TB]) may play a role. Suboptimal nutritional status and specific nutritional disorders (often subclinical) are particularly prevalent in the community.

For these reasons, the major limitation of this study was a lack of detailed information regarding patients’ medical history, including nutritional status. It is possible that some patients may have been suboptimally nourished or may have had systemic diseases not specifically mentioned on the referral form. On the other hand, in this respect the study population is likely to truly reflect what is present in the community.

## Conclusion

Although no method of SA estimation can be completely accurate, the findings of this study support the conclusion that the GP method should be used with caution in the South African population. Even given the relatively small sample size, the method was found to significantly underestimate age for both genders. On the other hand, in a few cases, overestimation is possible with individuals as young as 16 years old found by the method to have reached skeletal maturity. The findings of this study have significant clinical, forensic and medico-legal implications. Supplementary means of determination of SA should be considered, especially in medico-legal cases. Ultimately, accurate standards for SA estimation based on data from the South African population will need to be developed.
